# Rise and Growth of Vaccination Law.—II

**Published:** 1903-10-10

**Authors:** J. E. R. Stephens

**Affiliations:** Barrister-at-law, author of "Digest of Public Health Cases," etc., etc.


*(To be continued.)*

### RISE AND GROWTH OF VACCINATION LAW.—II.

By J. E. R. Stephens, Barrister-at-law, author of "Digest of Public Health Cases," etc., etc.

*(Continued from Vol. XXXIV., page 460.)*

Vaccination was made compulsory in Bavaria in 1807, and subsequently in the following countries:—Denmark (1810), Sweden (1814), the German States (1818), Russia (1835), Roumania (1874), Hungary (1876), and Servia (1881). It is compulsory by cantonal law in ten out of the twenty-two Swiss cantons. In the following countries there are no compulsory laws, but Government facilities and compulsion on various classes, more or less directly under Government control, such as soldiers, state employés, apprentices, school pupils, etc.:—France, Italy, Spain, Portugal, Belgium, Norway, Austria, Turkey. In only a few States of the American Union is there a vaccination statute; in Canada there is none. Vaccination has been compulsory in South Australia since 1872, in Victoria since 1874, and in Western Australia since 1878. In Tasmania a compulsory Act was passed in 1882. In New South Wales there is no compulsion, but free facilities for vaccination. Re-vaccination was compulsory in Denmark in 1871 and in Roumania in 1874; in Holland it was enacted for all school pupils in 1872. The various laws and administrative orders which had been for many years in force as to vaccination and re-vaccination in the several German states were consolidated in an imperial statute of 1874.

The first Vaccination Act in England was passed in 1840 (3 & 4 Vict. c. 29), and amended in 1841 by the 4 & 5 Vict. c. 32. This Act provided means of vaccination at the public cost, for every person in England and Wales, but left it entirely optional whether or not he would avail himself of its advantages. In 1853, the Act of 16 & 17 Vict. c. 100 was passed, which rendered vaccination compulsory. It directed guardians and overseers to divide their unions and parishes into districts for the purpose of affording increased facilities for the vaccination of the poor; it required stations to be established in each district, at which the medical officers should attend to perform the operation and inspect the result; and it imposed a penalty on parents and others having the care of children who, after notice, should fail to cause children under their control to be vaccinated, or when vaccinated to be taken to the vaccinator for inspection. The working of this Act not being found to be entirely satisfactory, the attention of the Government was called to the subject by a memorial addressed to the General Board of Health by the Epidemiological Society in 1855 (on a State provision for the prevention of small-pox and extension of vaccination, ordered by the House of Commons, to be printed March 1st 1855); and in 1856, an inquiry was instituted under the direction of the General Board of Health, the result of which was communicated to Parliament in 1857, accompanied by an introductory report by Mr. Simon, the medical officer of the board. Much valuable information as to the working of the law was also contained in the annual reports of the medical officer of the Privy Council; and eventually the subject was again brought under the attention of the Legislature.

In 1858 a further step was taken by the Legislature. The 21 and 22 Vict. c. 97, which was passed for one year only but was made perpetual by 22 and 23 Vict. c. 3, conferred on the Privy Council certain powers for promoting and superintending the execution of the Vaccination Acts. In 1861, by the 24 and 25 Vict. c. 59, guardians were authorised to appoint a person to institute and conduct proceedings for the purpose of enforcing obedience to the Acts; and power was given to justices to certify for expenses incurred by anyone so appointed, or by any registrar of births and deaths, or by any medical officer of health appointed under an Act of Parliament. The statute further declared that proceedings for enforcing penalties, on account of neglect to have a child vaccinated, might be taken at any time during which the parent or guardian of the child was in default. A difficulty, however, arose in enforcing penalties against a parent who, having been once proceeded against and fined, persisted in his refusal to have his child vaccinated. In *Pilcher* v. *Stafford* (33 L J.M.C. 113), an information was preferred against Stafford for neglecting within three months after the birth of his child to take it to one of the appointed medical officers for the purpose of being vaccinated. The defendant pleaded, in answer to the charge, that he had already been previously convicted upon a similar information, that he had been fined, and had paid the penalty and costs, and that therefore he was entitled to the protection extended to the persecuted by the old maxim, *nemo debet bis puniri pro uno delicto*. The case was argued before the Court of

Queen's Bench, and the Court held that when once the offence was complete and had been dealt with, and the person offending punished, further proceedings could not be taken against him under the existing law, although the child still remained unvaccinated.

In 1866 a Bill was brought before the House of Commons to consolidate and amend the law relating to vaccination. During the session of 1867 a similar Bill was again brought forward, and after reference to Select Committees of both Houses was passed, and received the Royal Assent on August 12th, 1867. This Act (30 and 31 Vict., c. 84) repealed all former Acts relating to vaccination, and required guardians to divide unions and parishes into vaccination districts, subject to the approval of the Poor Law Board (now Local Government Board), and to contract with some medical practitioner for the performance of vaccination within the district assigned to him. The qualification of the vaccinators was prescribed by the Privy Council, and the statute provided for special allowances to public vaccinators, and specified the minimum fees to be paid.

Provision was made for re-vaccination, for granting the necessary certificates, and for the payment of expenses incurred by the guardians in carrying the Act into force. The Act of 1867 forbade any attempt to produce small-pox by inoculation, specified the mode in which proceedings were to be taken, enabled the guardians to pay the reasonable costs of prosecutions undertaken by them or their officers, and directed that all such expenses should be charged to the common fund of the unions. The Vaccination Act of 1867 had not been long in operation before opposition to its provisions was raised and Parliament was petitioned to repeal it.

After the Vaccination Act of 1867 had been for three years in operation, it was found that considerable opposition to its compulsory provisions existed in some quarters; and for this and other reasons the Government, at the beginning of the session of 1871, proposed to the House of Commons to appoint a Select Committee to inquire into the operation of the Vaccination Act, 1867, and to report whether such Act should be amended. This proposal, on the motion of Mr. Forster, Vice-President of the Committee of the Council on Education, was agreed to by the House on February 13th, 1871, and on the 16th the Committee was appointed.

